# Association Between *Enterovirus* Infection and Type 1 Diabetes Risk: A Meta-Analysis of 38 Case-Control Studies

**DOI:** 10.3389/fendo.2021.706964

**Published:** 2021-09-07

**Authors:** Kan Wang, Fei Ye, Yong Chen, Jianxin Xu, Yufang Zhao, Yeping Wang, Tian Lan

**Affiliations:** ^1^Jinhua Maternity and Child Health Care Hospital, Jinhua, China; ^2^Jinhua Women and Children’s Hospital, Jinhua, China; ^3^First Department of Neurology, Affiliated Jinhua Hospital, Jinhua Municipal Central Hospital, Zhejiang University School of Medicine, Jinhua, China

**Keywords:** *enterovirus* infection, type 1 diabetes, case-control studies, odds ratio, meta-analysis

## Abstract

**Objective:**

The association between enterovirus infection and type 1 diabetes (T1D) is controversial, and this meta-analysis aimed to explore the correlation.

**Methods:**

PubMed, Embase, Web of Science, and Cochrane Database were searched from inception to April 2020. Studies were included if they could provide sufficient information to calculate odds ratios and 95% confidence intervals. All analyses were performed using STATA 15.1.

**Results:**

Thirty-eight studies, encompassing 5921 subjects (2841 T1D patients and 3080 controls), were included. The pooled analysis showed that enterovirus infection was associated with T1D (*P* < 0.001). Enterovirus infection was correlated with T1D in the European (*P* < 0.001), African (*P* = 0.002), Asian (*P* = 0.001), Australian (*P* = 0.011), and Latin American (*P* = 0.002) populations, but no conclusion could be reached for North America. The association between enterovirus infection and T1D was detected in blood and tissue samples (both *P* < 0.001); no association was found in stool samples.

**Conclusion:**

Our findings suggest that enterovirus infection is associated with T1D.

## Introduction

Type 1 diabetes (T1D) is a multifactorial disease resulting from the autoimmune destruction or dysfunction of pancreatic β cells (1). T1D has become a global burden, and at least 13 million individuals suffer from the disease worldwide (2, 3). Exogenous insulin injection cannot produce an optimal control of glucose homeostasis, leading to microvascular complications in the heart, brain, eye, kidney, and peripheral nervous system (4).

Although several environmental factors have been reported to be associated with T1D, enterovirus infection is under intensive focus (4–6). It is a ubiquitous, small, non-enveloped positive-strand RNA virus. *Enterovirus* genus belongs to the *Picornaviridae* family and consists of 15 species, seven of which contain human pathogens. These human infecting enteroviruses are classified into four species (Enterovirus A-D and Rhinovirus A-C) and contain more than 250 serologically distinct viruses. Enterovirus A-D consists of over 100 different types, including polioviruses, coxsackievirus types A and B (CVA and CVB), numbered enteroviruses, and echoviruses (7, 8). Enteroviruses potentially interact with several receptors (9), among which the coxsackie and adenovirus receptor (CAR) is the most studied with respect to T1D. Enteroviruses can infect pancreatic β cells in pancreatic islets *via* the CAR, which is expressed on β and α cells, and the viruses replicate in both these cell types (10, 11). Both acute and persistent enterovirus infections have been shown to affect the functions of the host cell, inducing β cell death, decreasing insulin mRNA expression and insulin secretion, and disrupting the Golgi apparatus (11–16).

A meta-analysis identified the correlation between enterovirus infection and T1D in 2011 (17). Although several original studies have been reported from 2012 to 2020 (10, 18–34), no updated meta-analysis has been performed to explore and refine the correlation. The conclusions of the original studies are conflicting, and thus, a meta-analysis including the latest studies is still needed to evaluate the association between enterovirus infection and T1D.

## Materials and Methods

### Search Strategy

PubMed, Embase, Web of Science, and Cochrane Database were searched for relevant studies. We used the following terms for searching: “enterovirus” AND (“type 1 diabetes” OR “type 1 diabetic patients” OR “type 1 diabetes mellitus” OR “insulin-dependent diabetes” OR “insulin-dependent diabetic patients” OR “T1D” OR “T1DM”). The searches were restricted to English-language articles published up to April 2020. We also reviewed the references of included articles to identify any potential additional study.

### Inclusion and Exclusion Criteria

The studies were eligible if they met the following criteria: (1) study design: case-control; (2) outcomes: investigated the association between enterovirus infection and T1D and reported the number of subjects with and without enterovirus infection for each group; (3) subjects: patients with insulin-dependent diabetes (i.e., T1D); and (4) controls: non-diabetic individuals. When there were multiple publications from the same study population, only the publication with the largest sample size was included. Studies were excluded if they were (1) reviews, letters, or case reports, (2) cell or animal studies, or (3) duplicate publications from the same population.

### Data Extraction

Data were extracted independently by two authors. Disagreements were resolved by a third author. The following information was extracted: first author, publication year, country, mean age, the gender ratio of the cases, number of patients in the case and control group, number of enterovirus infections in each group, detection method, sample source, and enterovirus type. We also contacted the corresponding author to obtain details of the missing relevant data.

### Quality Assessment

The Newcastle-Ottawa quality assessment scale (NOS) (35), a 9-star system, was used for quality assessment. Two authors assessed the studies independently. Any differences were resolved by consulting a third author. The assessment scale included the selection method of the exposed group (with enterovirus infection) and the non-exposed group (without enterovirus infection), the matching of the two groups, and the outcome assessment. A study awarded more than 5 stars was considered a high-quality study.

### Statistical Analysis

Odds ratio (OR) and 95% confidence interval (CI) were used to estimate the strength of the association between enterovirus infection and T1D. The fixed-effect model was used for non-heterogeneous data, and the random effect model was used for heterogeneous data. The Q and *I* ^2^ statistics were used to test for heterogeneity. If statistically significant heterogeneity was present (Q statistic *P* < 0.05 or *I* ^2^ ≥ 50%), the random-effect model was applied; otherwise, the fixed-effect model was used (36). In order to explore the potential sources of heterogeneity, we conducted subgroup analyses by continents (Asia, Europe, North America, or Africa), detection methods (PCR, ELISA, or immunostaining), sample sources (blood, tissue, or stool), and study quality (NOS score ≥ 6 or < 6). The sensitivity analysis was conducted by the sequential removal of each study. Begg’s correlation and Egger’s regression were used to assess the potential publication bias (37, 38). All analyses were conducted using STATA 15.1 (Stata, College Station, TX, USA).

## Results

### Characteristics of the Studies Included in the Meta-Analysis

**Figure 1:**
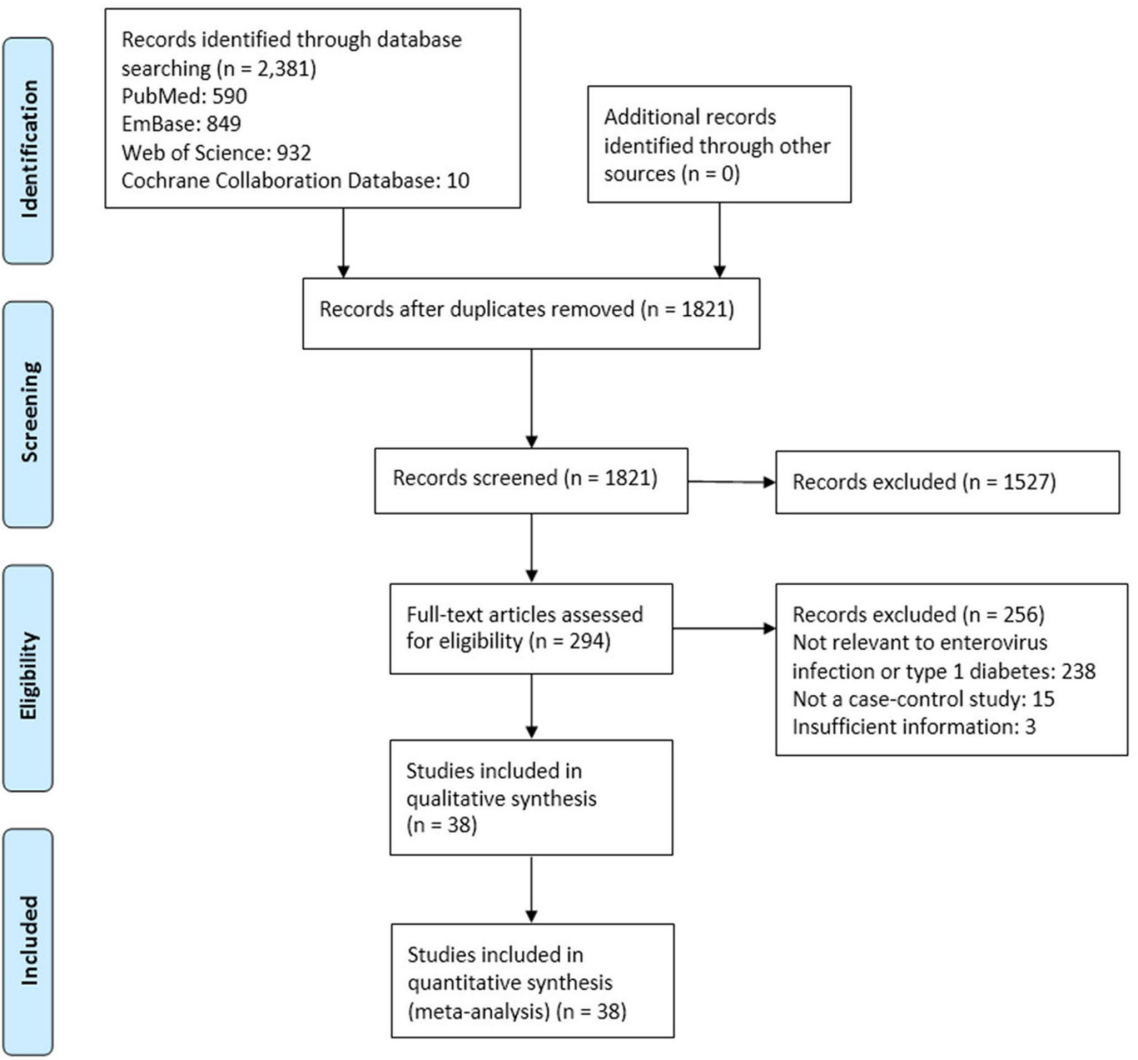
Schematic of the process of selecting studies for the meta-analysis.

The study process is shown in **Figure 1**. Among 1501 potentially relevant studies, 38 met the inclusion criteria (10, 18–34, 39–58). The dataset included 5921 subjects (2841 T1D patients and 2841 controls). The included studies were published from 1990 to 2019, with sample sizes ranging from 7 to 766. Of these studies, 25 were from Europe, four from Africa, two from Asia, two from Australia, one from North America, and one from Latin America. Most studies were in Caucasians. No study was excluded due to poor quality. Detailed information of all the included studies is listed in **Table 1**. The results of the quality evaluation are shown in **Supplement Table 1**.

**Table 1:** Characteristics of the 38 studies included in the present meta-analysis.

Author, publication year	Country	Ethnicity	Mean age of cases (year)	Male of cases (%)	No. of case/control	No. of EV infection (case/control)	Detection method	EV type	Sample source	NOS scale
Takita (18)	Japan	Asian	22.7	100.0	3/17	3/0	Immunostaining	VP1	tissue	6
Kim (19)	Sydney	Mixed	5.7	56.0	45/48	11/5	RT-PCR	EV-A, EV-B	blood	5
Vehik (20)	USA	Mixed	–	–	383/383	78/76	RT-PCR	CVB	stool	6
Zargari (21)	Iran	Caucasian	13.7	–	35/35	10/0	ELISA	VP1	blood	6
Federico (22)	Italy	Caucasian	9.4	46.3	82/117	53/0	Immunostaining and virus culture in cells followed by end-point PCR (59)	–	blood	5
Nekoua (23)	Benin	African	21.8	40.0	15/8	11/2	ELISA	PV1, CVB-4	saliva or blood	7
El-Senousy (24)	Egypt	African	9.8	50.0	382/100	100/0	RT-PCR	CVB-4	blood	5
Karaoglan (25)	Turkey	Caucasian	8.2	57.5	40/30	3/0	Serology	IVB, ECHO7, PIV4, CAV7, H3N2 CBV4	blood	6
Aida (26)	Japan	Asian	61.5	41.7	12/19	0/0	Immunostaining	VP1	blood	4
Honkanen (27)	Finland	Caucasian	11.0	–	97/221	50/86	RT-PCR	CVA, CVB, ECHO, EV-68, EV-71, EV-90	stool	6
Boussaid (28)	Tunisia	Afican	19.7	61.1	95/141	30/11	RT-PCR	–	blood	7
Abdel-Latif (29)	Egypt	African	9.8	60.0	382/100	100/0	RT-PCR	–	blood	7
Hodik (10)	Sweden	Caucasian	–	–	27/24	15/6	RT-PCR	–	tissue	5
Krogvold (30)	Norway	Caucasian	28.8	50.0	6/6	4/0	Immunostaining (VP1) and RT-PCR	VP1	tissue	4
Laitinen (31)	Finland	Caucasian	–	–	183/366	108/183	Neutralization assay	CVB-1	blood	5
Cinek (32)	Norway	Caucasian	–	–	45/92	11/25 blood samples	RT-PCR	–	blood	6
Salvatoni (33)	Italy	Caucasian	9.7	62.5	24/26	19/0	Virus culture in cells followed by end-point PCR (59)	–	blood	6
Oikarinen (34)	Finland	Caucasian	43.0	28.2	39/41	29/12	Immunostaining	–	tissue	5
Schulte (39)	Netherlands	Caucasian	9.7	50.0	10/20	4/0	RT-PCR	HEV-B	blood	4
Richardson (40)	UK	Caucasian	12.7	–	72/119	44/12	Immunostaining	–	tissue	4
Dotta (41)	Italy	Caucasian	13.8	33.3	6/26	3/0	Immunostaining	CVB-4	tissue	5
Oikarinen (42)	Finland	Caucasian	32.7	16.7	12/10	6/0	RT-PCR	–	tissue	4
Sarmiento (43)	Cuba	Mixed	7.3	38.2	34/68	9/2	RT-PCR	–	blood	6
Moya-Suri (44)	Germany	Caucasian	13.0	51.1	47/50	17/2	RT-PCR	CVB-4, CVB-2, CVB-6	blood	7
Salminen (45)	Finland	Caucasian	12.3	41.7	12/53	10/22	RT-PCR	PV-3, CVA-9, CVB-3, CVB-4, CVB-5, EV-3, EV-11, EV-18, EV-24, EV-25	blood	7
Ylipaasto (46)	Finland/Germany	Caucasian	–	40.0	65/40	4/0	RT-PCR	–	tissue	5
Craig (47)	Australia	Mixed	8.1	38.3	206/160	62/6	RT-PCR	EV-71	blood or stool	6
Sadeharju (48)	Finland	Caucasian	–	–	19/84	3/7	RT-PCR	CVB-4, EV-11	blood	8
Salminen (49)	Finland	Caucasian	–	53.7	41/196	7/8	RT-PCR	CVB-4, EV-11	blood	6
Coutant (50)	France	Caucasian	–	–	16/49	2/1	RT-PCR	–	blood	6
Yin (51)	Sweden	Caucasian	8.6	75.0	24/24	18/7	RT-PCR	CVB-5, EV-5, CVB-4	blood	7
Lönnrot (52)	Finland	Caucasian	8.4	61.0	49/105	11/2	RT-PCR	–	blood	6
Nairn (53)	UK	Caucasian	7.1	–	110/182	30/9	RT-PCR	PV1-3, CVA-21, CVA-24, EV-70	blood	7
Andréoletti (54)	France	Caucasian	28.2	50.0	12/15	5/0	RT-PCR	CVB-3, CVB-4	blood	4
Clements (55)	UK	Caucasian	3.9	–	14/45	9/2	RT-PCR	CVB-3, CVB-4	blood	6
Foy (56)	UK	Caucasian	11.0	58.2	55/42	22/13	RT-PCR	–	blood	6
Buesa-Gomez (57)	USA	Mixed	8.5	50.0	2/5	0/0	RT-PCR	–	tissue	4
Foulis (58)	UK	Caucasian	–	–	147/43	0/0	Immunostaining	–	tissue	3

EV, enterovirus; RT-PCR, reverse transcription-polymerase chain reaction; ELISA, enzyme-linked immunosorbent assay; PV1, poliovirus type 1; CVB, Coxsackie virus B; VP1, enterovirus capsid protein 1; NOS, Newcastle–Ottawa quality assessment scale.

### Pooled Analysis

**Figure 2:**
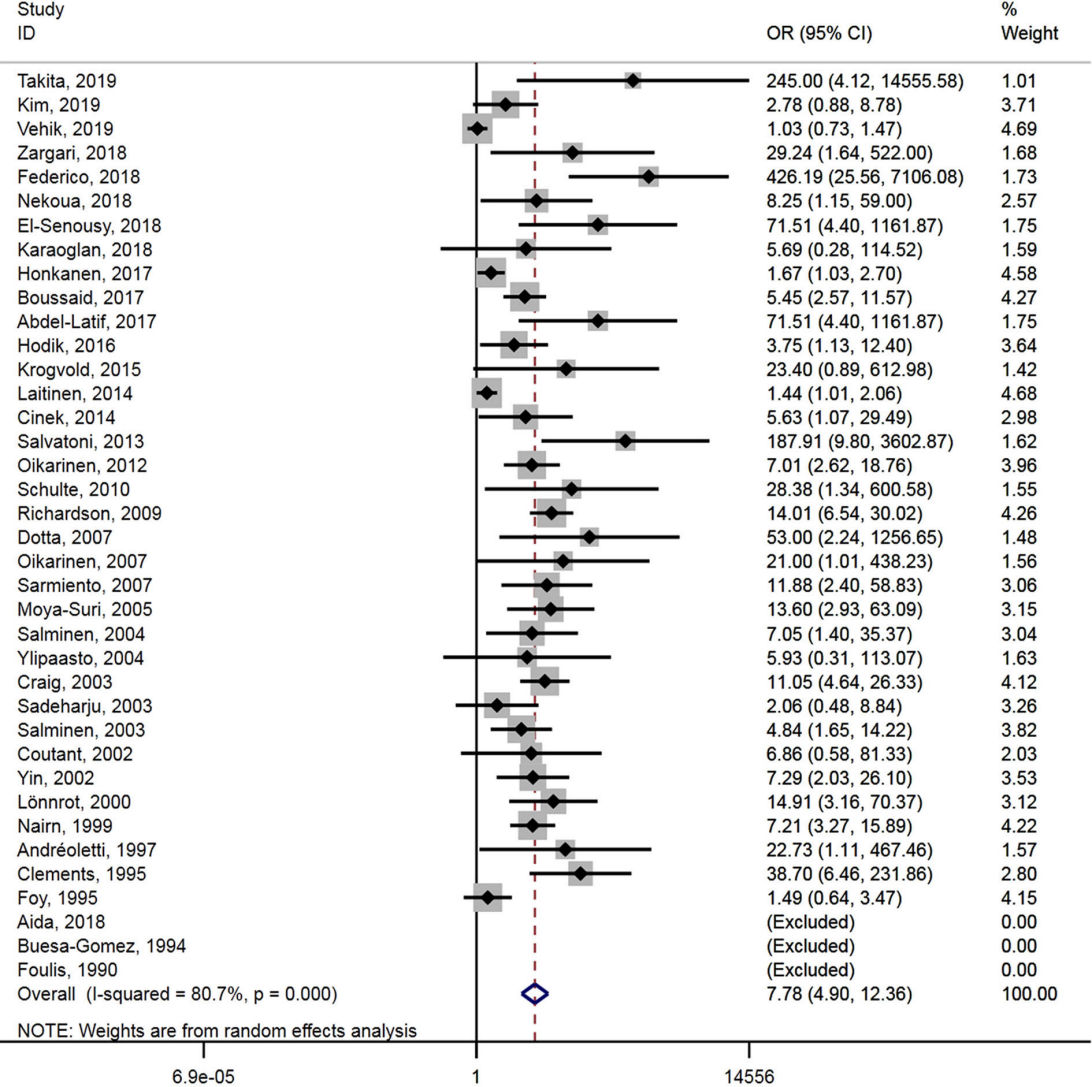
Forest plot of ORs of enterovirus and type 1 diabetes.

A total of 38 studies reported the association between enterovirus infection and T1D. Enterovirus infection was associated with T1D (OR = 7.8, 95% CI = 4.9–12.4, *P* < 0.001) (**Figure 2**), and substantial heterogeneity was observed among the studies (*P* < 0.001, *I* ^2^ = 80.7%).

### Subgroup Analysis

Studies were categorized by continent, detection method, sample source, and study quality in the subgroup analysis. Enterovirus infection was correlated with T1D in the European (OR = 7.5, 95% CI = 4.4–12.6, *P* < 0.001), African (OR = 16.5, 95% CI = 2.8–95.1, *P* = 0.002), Asian (OR = 245.0, 95% CI = 4.1–15000.0, *P* = 0.001), Australian (OR = 5.8, 95% CI = 1.5–22.9, *P* = 0.011), and Latin American (OR = 11.9, 95% CI = 2.4–58.8, *P* = 0.002) populations. The study from North America reported no association between enterovirus and T1D, but since only one study was included, no conclusion could be reached. The association between enterovirus infection and T1D was shown in blood samples (OR = 8.8, 95% CI = 4.9–15.9, *P* < 0.001) and tissue samples (OR = 9.9, 95% CI = 5.5–17.8, *P* < 0.001), but none was detected in stool samples. Furthermore, no significant difference was observed between different detection methods and study quality (**Table 2**).

**Table 2:** Subgroup analysis results.

Subgroup		Number of studies	OR	95% CI		*P*	Heterogeneity	
				Lower	Upper		*P*	*I^2^* (%)
Continent								
Europe		25	7.5	4.4	12.6	<0.001	<0.001	76.4
Africa		4	16.5	2.8	95.1	0.002	0.018	70.2
Asia		2	245.0	4.1	15,000.0	0.001	0.379	0.0
Australia		2	5.8	1.5	22.9	0.011	0.057	72.3
North America		1	1.0	0.7	1.5	0.857	NA	NA
Latin America		1	11.9	2.4	58.8	0.002	NA	NA
Detection method								
RT-PCR		26	6.8	4.1	11.4	<0.001	<0.001	78.1
Immunostaining		7	15.1	3.5	65.5	<0.001	<0.001	89.7
ELISA		2	12.3	2.4	62.6	0.002	0.449	0.0
Sample source								
Blood		23	8.8	4.9	15.9	<0.001	<0.001	76.9
Tissue		8	9.9	5.5	17.8	<0.001	0.344	11.1
Stool		2	1.3	0.8	2.0	0.307	0.115	59.8
Study quality								
NOS score ≥6		22	6.9	3.9	12.1	<0.001	<0.001	80.3
NOS score <6		13	11.2	4.3	29.4	<0.001	<0.001	82.9

RT-PCR, reverse transcription-polymerase chain reaction; ELISA, enzyme-linked immunosorbent assay; OR, odds ratio; CI, confidence interval.

### Sensitivity Analysis

**Figure 3:**
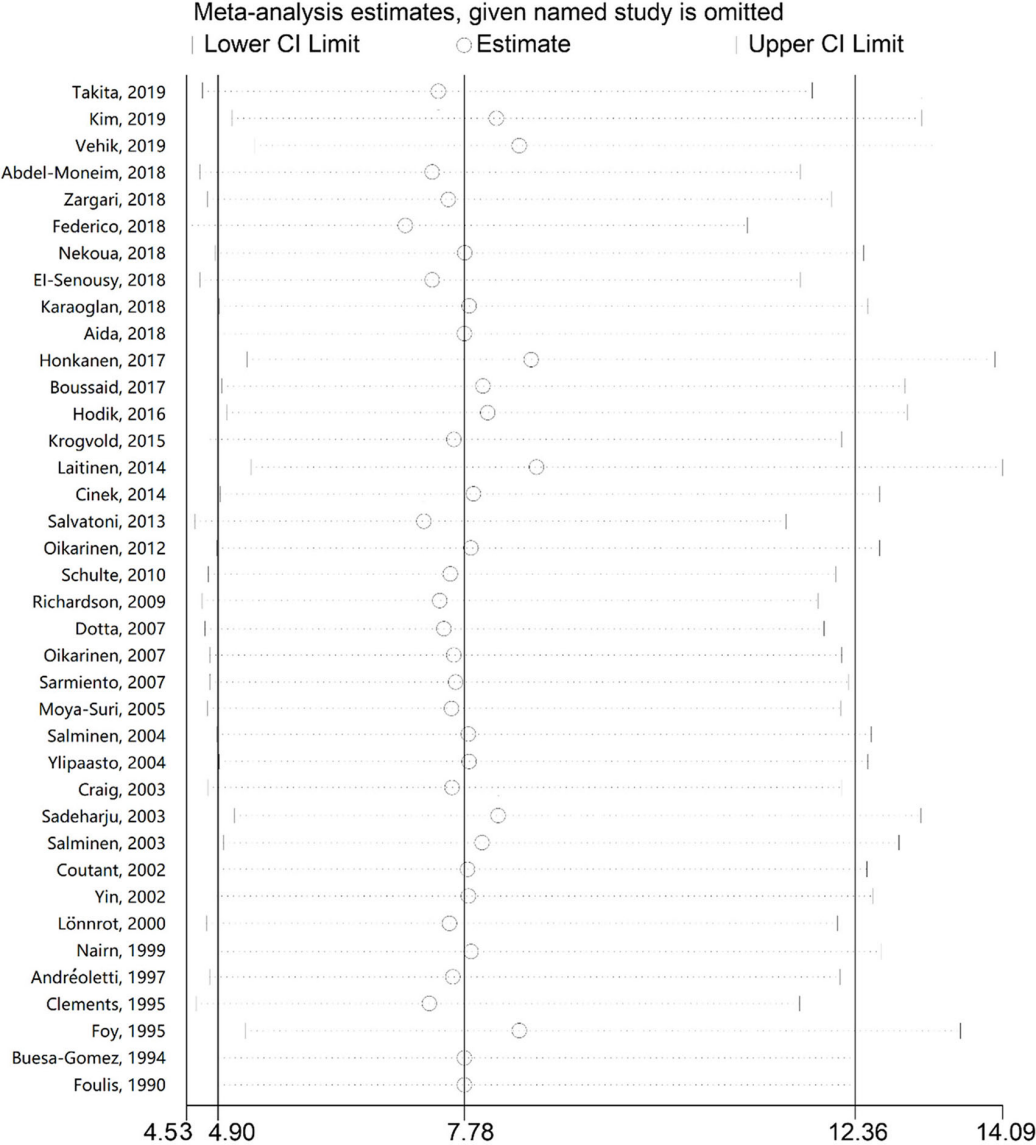
Sensitivity analysis of the association between enterovirus and type 1 diabetes. The odds ratios and 95% confidence intervals (CIs) for the association between enterovirus and type 1 diabetes were recalculated by sequentially excluding each study indicated on the left.

In order to evaluate the influence of each study on the pooled OR, the sensitivity analysis was performed by the sequential removal of every study. The results showed no significant variation in OR, which reflected the stability and robustness of our results (**Figure 3**).

### Publication Bias

Funnel plots showed a slight asymmetry. Publication bias was indicated by P-values from Egger’s regression (*P* < 0.001); however, no significant publication bias was indicated by P-values from Begg’s test (*P* = 0.151).

## Discussion

The incidence of T1D is rising in many countries. Environmental factors, especially enterovirus infection, might be involved in the initiation and acceleration of the pathogenesis of T1D (60). Although a previous meta-analysis was conducted to identify whether enterovirus infection was associated with T1D (17), the present meta-analysis consisted of the largest number of original studies and subjects available to evaluate the association. Furthermore, we conducted a subgroup analysis of the detection method and sample source, which was not performed in the previous meta-analysis.

In the present study, 38 case-control studies, consisting of 5921 subjects (2841 T1D subjects and 3080 controls) were included. The pooled analysis showed that enterovirus infection is associated with T1D, with almost 8-fold the odds of enterovirus infection in T1D compared with the controls, consistent with the previous meta-analysis (17). As the new studies included Asian and African populations, a finding of significant association in these populations suggests that the correlation with relatively high T1D rates found in European populations is also observed in other populations. Karaoglan et al. (25) investigated the serologic epidemiological and molecular evidence on enteroviruses and respiratory viruses in patients with newly-diagnosed T1D during the cold season and showed that enteroviruses and respiratory viruses, in addition to seasonal infections, could play a role in the etiopathogenesis and clinical onset of T1D. Honkanen et al. (27) evaluated whether the presence of enterovirus was associated with the appearance of islet autoimmunity in T1D and found that enterovirus infection diagnosed by detecting viral RNA was associated with the development of islet autoimmunity with an interval of several months. In the subgroup analysis, enterovirus infection was correlated with T1D in Europe, Africa, Asia, Australia, and Latin American, but no conclusion could be reached for North America. Moreover, the association between enterovirus infection and T1D was shown in blood and tissue samples, but no association was detected in stool samples, possibly because only two studies presented data from stool specimens and because stool sampling and handling are subject to more technical variability than blood, for example, especially if stool sampling is performed at home. Thus, the subgroup variability needs to be investigated in future studies. Sensitivity analysis showed that this meta-analysis results were robust, without a single study influencing the results by itself, indicating statistical stability and reliability. Still, a significant publication bias was observed in Egger’s test, suggesting a possible under-reporting of negative results or no reports from smaller centers with less experience.

Enterovirus infection is associated with the destruction of β cells (1). Two recent studies have shown that CVB1 is associated with an increased risk of β-cell autoimmunity, while CVB3 and CVB6 are associated with a reduced T1D risk (31, 61). CVB1 has been reported to infect human pancreatic islets *in vitro*; it is one of the most cytolytic enterovirus serotypes in this model (62). In addition, an *in vivo* study performed in CBS/j mice demonstrated that the CVB3 virus did not affect glucose tolerance, while CVB4 did (63). Only a few original studies in our meta-analysis have provided the enterovirus type for cases and controls, and hence, we could not establish the correlation between enterovirus type and T1D. Still, those studies (31, 61–63) suggest that different strains of enteroviruses could have different impacts on the development of T1D through variations in the genome of the viruses. One of the limitations in all these studies is the difficulty of obtaining not just the evidence of serotype but the complete enterovirus genomes from human patients at the time of T1D diagnosis. In addition, obtaining the complete genomes from stool samples is technically difficult. Future studies will have to examine more closely the strains associated with T1D as well as the genomes and mechanisms involved since the development of T1D might vary with serotypes.

Some limitations should be noted. First, the sample size is still small in this meta-analysis, especially in the subgroup analysis. Second, although some of the original studies detected the enterovirus types, most of them did not provide the number of T1D patients per enterovirus type. Therefore, we could not examine the correlation between enterovirus type and T1D. Third, although subgroup and sensitivity analyses were conducted, a source of heterogeneity was still not found, which could be attributed to the insufficient information obtained from the original studies. Fourth, the further evaluation of potential gene-gene or gene-environment interactions was limited by the insufficient original data. Despite the limitations, our meta-analysis significantly increased the statistical power based on substantial data from different studies.

## Conclusion

Our findings suggest that enterovirus infection is associated with T1D. This study might provide a scientific basis for identifying the infectious agents associated with T1D and for the possible prevention of T1D through vaccines and other means. Studies with a larger sample size, especially from the US and China, are needed to reach a definitive conclusion.

## Data Availability Statement

The original contributions presented in the study are included in the article/**Supplementary Material**. Further inquiries can be directed to the corresponding authors.

## Author Contributions

KW and FY, study design and manuscript writing. YC, data collection and data analysis. JX, data interpretation. YZ, preparation of the manuscript. YW and TL, literature analysis. All authors contributed to the article and approved the submitted version.

## Funding

This work was supported by the Jinhua Science and Technology Bureau (Grant no.2020-3-036).

## Conflict of Interest

The authors declare that the research was conducted in the absence of any commercial or financial relationships that could be construed as a potential conflict of interest.

## Publisher’s Note

All claims expressed in this article are solely those of the authors and do not necessarily represent those of their affiliated organizations, or those of the publisher, the editors and the reviewers. Any product that may be evaluated in this article, or claim that may be made by its manufacturer, is not guaranteed or endorsed by the publisher.

## Abbreviations

T1D, type 1 diabetes; CAR, coxsackie and adenovirus receptor; NOS, Newcastle-Ottawa quality assessment scale; OR, odds ratio; CI, confidence interval; CVA, coxsackievirus type A; CVB, coxsackievirus type B.
