# Supplementary material for: Association Between Enterovirus Infection and Type 1 Diabetes Risk: A Meta-Analysis of 38 Case-Control Studies
Source: Front Endocrinol (Lausanne). 2021 Sep 7;12:706964. doi: 10.3389/fendo.2021.706964 (PMC8453141; doi:10.3389/fendo.2021.706964)
Supplement: Supplementary file 1 [file Table_1.doc]

Supplement Table 1. Assessment of study quality using the Newcastle–Ottawa Scale

| First author, publication year | Diagnosis of case group | Representativeness of the case group | Selection of the control group | Definition of the control group | Comparability of groups on the basis of the design or analysis | Assessment of exposure | Assessment methods of groups same | No response rate | Total |
| --- | --- | --- | --- | --- | --- | --- | --- | --- | --- |
| [Takita](https://www.ncbi.nlm.nih.gov/pubmed/?term=Takita%20M%5BAuthor%5D&cauthor=true&cauthor_uid=31112279), 2019 ([15](#_ENREF_15)) | ☆ | - | ☆ | ☆ | ☆ | ☆ | ☆ | - | 6 |
| Kim, 2019 ([16](#_ENREF_16)) | ☆ | - | - | ☆ | ☆ | ☆ | ☆ | - | 5 |
| [Vehik](https://www.ncbi.nlm.nih.gov/pubmed/?term=Vehik%20K%5BAuthor%5D&cauthor=true&cauthor_uid=31792456), 2019 ([17](#_ENREF_17)) | - | ☆ | - | ☆ | ☆☆ | ☆ | ☆ | - | 6 |
| Zargari, 2018 ([18](#_ENREF_18)) | ☆ | - | - | ☆ | ☆☆ | ☆ | ☆ | - | 6 |
| [Federico](https://www.ncbi.nlm.nih.gov/pubmed/?term=Federico%20G%5BAuthor%5D&cauthor=true&cauthor_uid=29569355), 2018 ([19](#_ENREF_19)) | ☆ | - | - | ☆ | ☆ | ☆ | ☆ | - | 5 |
| Nekoua, 2018 ([20](#_ENREF_20)) | ☆ | - | ☆ | ☆ | ☆☆ | ☆ | ☆ | - | 7 |
| [El-Senousy](https://www.ncbi.nlm.nih.gov/pubmed/?term=El-Senousy%20WM%5BAuthor%5D&cauthor=true&cauthor_uid=29022248), 2018 ([21](#_ENREF_21)) | ☆ | - | - | ☆ | ☆ | ☆ | ☆ | - | 5 |
| [Karaoglan](https://www.ncbi.nlm.nih.gov/pubmed/?term=Karaoglan%20M%5BAuthor%5D&cauthor=true&cauthor_uid=30186878), 2018 ([22](#_ENREF_22)) | - | - | ☆ | ☆ | ☆☆ | ☆ | ☆ | - | 6 |
| Aida, 2018 ([23](#_ENREF_23)) | - | ☆ | - | ☆ | - | ☆ | ☆ | - | 4 |
| [Honkanen](https://www.ncbi.nlm.nih.gov/pubmed/?term=Honkanen%20H%5BAuthor%5D&cauthor=true&cauthor_uid=28070615), 2017 ([24](#_ENREF_24)) | ☆ | ☆ | - | ☆ | ☆ | ☆ | ☆ | - | 6 |
| [Boussaid](https://www.ncbi.nlm.nih.gov/pubmed/?term=Boussaid%20I%5BAuthor%5D&cauthor=true&cauthor_uid=29176023), 2017 ([25](#_ENREF_25)) | ☆ | - | - | ☆ | ☆☆ | ☆ | ☆ | ☆ | 7 |
| Abdel-Latif, 2017 ([26](#_ENREF_26)) | ☆ | ☆ | - | ☆ | ☆☆ | ☆ | ☆ | - | 7 |
| Hodik, 2016 ([10](#_ENREF_10)) | - | - | ☆ | ☆ | ☆ | ☆ | ☆ | - | 5 |
| Krogvold, 2015 ([27](#_ENREF_27)) | ☆ | - | - | ☆ | - | ☆ | ☆ | - | 4 |
| [Laitinen](https://www.ncbi.nlm.nih.gov/pubmed/?term=Laitinen%20OH%5BAuthor%5D&cauthor=true&cauthor_uid=23974921), 2014 ([28](#_ENREF_28)) | - | ☆ | - | ☆ | ☆ | ☆ | ☆ | - | 5 |
| [Cinek](https://www.ncbi.nlm.nih.gov/pubmed/?term=Cinek%20O%5BAuthor%5D&cauthor=true&cauthor_uid=25047648), 2014 ([29](#_ENREF_29)) | ☆ | ☆ | - | ☆ | ☆ | ☆ | ☆ | - | 6 |
| [Salvatoni, 2013](https://www.ncbi.nlm.nih.gov/pubmed/?term=Salvatoni%20A%5BAuthor%5D&cauthor=true&cauthor_uid=23763622)([30](#_ENREF_30)) | - | - | ☆ | ☆ | ☆☆ | ☆ | ☆ | - | 6 |
| [Oikarinen](https://www.ncbi.nlm.nih.gov/pubmed/?term=Oikarinen%20M%5BAuthor%5D&cauthor=true&cauthor_uid=22315304), 2012 ([31](#_ENREF_31)) | ☆ | - | - | ☆ | ☆ | ☆ | ☆ | - | 5 |
| Schulte, 2010 ([36](#_ENREF_36)) | - | ☆ | - | ☆ | - | ☆ | ☆ | - | 4 |
| Richardson, 2009 ([37](#_ENREF_37)) | ☆ | - | - | ☆ | - | ☆ | ☆ | - | 4 |
| Dotta, 2007 ([38](#_ENREF_38)) | ☆ | ☆ | - | ☆ | - | ☆ | ☆ | - | 5 |
| Oikarinen, 2007 ([39](#_ENREF_39)) | - | ☆ | - | ☆ | - | ☆ | ☆ | - | 4 |
| Sarmiento, 2007 ([40](#_ENREF_40)) | ☆ | ☆ | - | ☆ | ☆ | ☆ | ☆ | - | 6 |
| Moya-Suri, 2005 ([41](#_ENREF_41)) | - | ☆ | - | ☆ | ☆☆ | ☆ | ☆ | ☆ | 7 |
| Salminen, 2004 ([42](#_ENREF_42)) | ☆ | - | - | ☆ | ☆☆ | ☆ | ☆ | ☆ | 7 |
| Ylipaasto, 2004 ([43](#_ENREF_43)) | - | - | - | ☆ | ☆☆ | ☆ | ☆ | - | 5 |
| Craig, 2003 ([44](#_ENREF_44)) | ☆ | ☆ | ☆ | ☆ | - | ☆ | ☆ | - | 6 |
| Sadeharju, 2003 ([45](#_ENREF_45)) | ☆ | ☆ | - | ☆ | ☆☆ | ☆ | ☆ | ☆ | 8 |
| Salminen, 2003 ([46](#_ENREF_46)) | ☆ | - | - | ☆ | ☆☆ | ☆ | ☆ | - | 6 |
| Coutant, 2002 ([47](#_ENREF_47)) | - | ☆ | - | ☆ | ☆☆ | ☆ | ☆ | - | 6 |
| Yin, 2002 ([48](#_ENREF_48)) | ☆ | - | - | ☆ | ☆☆ | ☆ | ☆ | ☆ | 7 |
| Lönnrot, 2000 ([49](#_ENREF_49)) | ☆ | ☆ | - | ☆ | ☆ | ☆ | ☆ | - | 6 |
| Nairn, 1999 ([50](#_ENREF_50)) | ☆ | - | ☆ | ☆ | ☆ | ☆ | ☆ | ☆ | 7 |
| Andréoletti, 1997 ([51](#_ENREF_51)) | - | ☆ | - | ☆ | - | ☆ | ☆ | - | 4 |
| Clements, 1995 ([52](#_ENREF_52)) | ☆ | - | - | ☆ | ☆☆ | ☆ | ☆ | - | 6 |
| Foy, 1995 ([53](#_ENREF_53)) | - | ☆ | - | ☆ | ☆☆ | ☆ | ☆ | - | 6 |
| Buesa-Gomez, 1994 ([54](#_ENREF_54)) | ☆ | - | - | ☆ | - | ☆ | ☆ | - | 4 |
| Foulis, 1990 ([55](#_ENREF_55)) | - | - | - | ☆ | - | ☆ | ☆ | - | 3 |
